# Use of Whole-Genome Sequencing to Link *Burkholderia pseudomallei* from Air Sampling to Mediastinal Melioidosis, Australia

**DOI:** 10.3201/eid2111.141802

**Published:** 2015-11

**Authors:** Bart J. Currie, Erin P. Price, Mark Mayo, Mirjam Kaestli, Vanessa Theobald, Ian Harrington, Glenda Harrington, Derek S. Sarovich

**Affiliations:** Royal Darwin Hospital, Darwin, Northern Territory, Australia (B.J. Currie);; Menzies School of Health Research, Casuarina, Northern Territory (B.J. Currie, E.P. Price, M. Mayo, M. Kaestli, V. Theobald, I. Harrington, G. Harrington, D.S. Sarovich)

**Keywords:** melioidosis, *Burkholderia pseudomallei*, pneumonia, gram-negative bacterial infections, pathogen transmission, biothreat, bacteria, zoonoses, genome, Australia

## Abstract

The frequency with which melioidosis results from inhalation rather than percutaneous inoculation or ingestion is unknown. We recovered *Burkholderia pseudomallei* from air samples at the residence of a patient with presumptive inhalational melioidosis and used whole-genome sequencing to link the environmental bacteria to *B. pseudomallei* recovered from the patient.

Melioidosis is thought to be caused predominantly by percutaneous inoculation with the bacterium *Burkholderia pseudomallei*; however, inhalation, aspiration, and ingestion of the bacterium can also occur (*1*). Although an evidence-based clinical definition of inhalational melioidosis has recently been published (*2*), the proportion of melioidosis cases resulting from inhalation is unknown, and attempts to culture *B. pseudomallei* from air sampling in melioidosis-endemic regions have been largely unsuccessful (*3*). We recovered *B. pseudomallei* from air samples at the residence of a patient with presumptive inhalational melioidosis, and whole-genome sequencing linked the environmental bacteria to *B. pseudomallei* recovered from the patient.

## The Study

**Figure 1:**
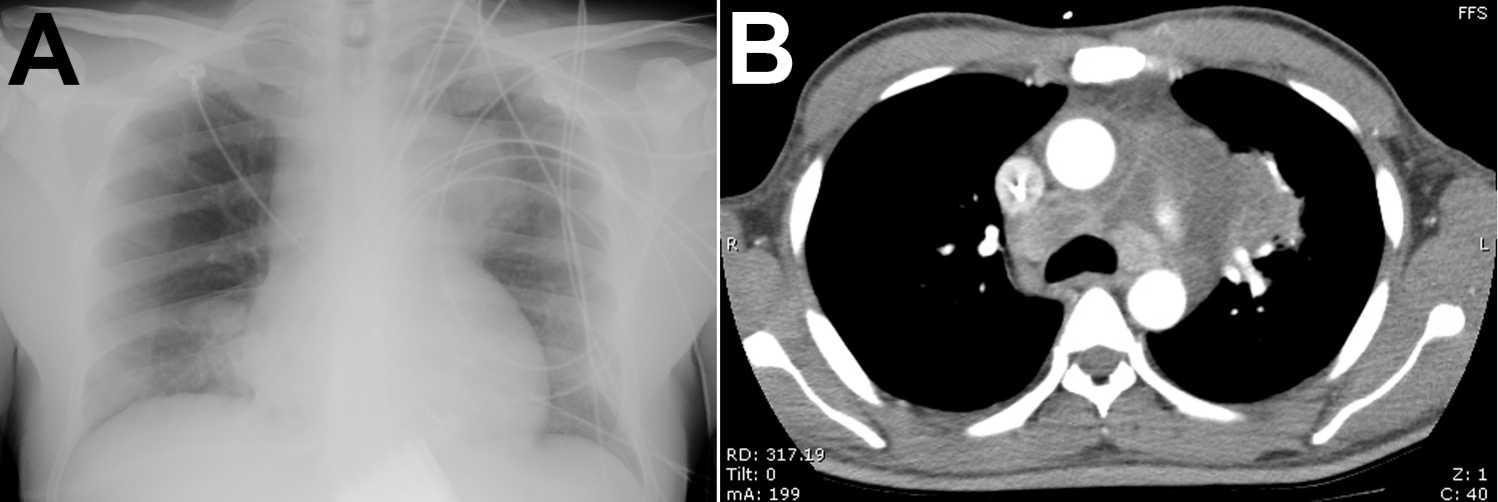
Clinical studies of a patient with melioidosis, Royal Darwin Hospital, Darwin, Northern Territory, Australia. A) Chest radiograph shows a soft-tissue mass associated with the left side of the mediastinum and obscuring the aortic arch. B) Chest computed tomography scan shows a large loculated mass in the anterior mediastinum; the mass is contiguous with multiple enlarged mediastinal lymph nodes and with pulmonary consolidation.

A 47-year-old man with poorly controlled type 2 diabetes sought care at the Royal Darwin Hospital in the tropical north of the Northern Territory of Australia in January 2011 after several weeks of increasing lethargy and 1 week of fevers and cough. He was patient 692 in the Darwin Prospective Melioidosis Study (*4*), which is approved by the Human Research Ethics Committee of the Northern Territory Department of Health and the Menzies School of Health Research (approval 02/38). The patient’s chest radiograph (Figure 1, panel A) showed patchy pneumonia in the right lung and a large soft-tissue mass on the left side of the chest. A computed tomography scan (Figure 1, panel B) confirmed the mass to be a 7-cm × 6-cm loculated fluid collection in the anterior mediastinum contiguous with patchy bilateral pneumonia and associated with multiple enlarged mediastinal lymph nodes and a pericardial effusion. Blood cultures collected at admission were positive for *B. pseudomallei*. The patient required initial management in the intensive care unit for his pneumonia, severe sepsis, and ketoacidosis. He received 45 days of intravenous antimicrobial drugs (meropenem for 11 days, followed by ceftazidime for 34 days) in conjunction with oral trimethoprim/sulfamethoxazole. After discharge, he received 15 subsequent weeks of eradication therapy with trimethoprim/sulfamethoxazole. At follow up, he has remained well 4 years after completing his melioidosis therapy.

The unusual finding of extensive mediastinal disease raised the possibility that the patient had inhalational melioidosis. The patient described sitting most days outside his urban accommodation on an elevated and exposed mowed grassy area that overlooks ground sloping downhill to a rocky open drain, an environment that prompted the potential for targeted air sampling. The site was visited and environmental samples taken 6 weeks after the patient’s hospital admission. During the sampling, squally rain showers occurred, accompanied by wind blowing up the hill and the drain flowing swiftly.

Two air samplings and 3 soil samples were collected. Each air collection entailed passing 1,000 L of air (50 L/min for 20 min) through a portable microbiologic air sampler (MD8 AirPort; Sartorius Stedim, Dandenong, Victoria, Australia) with a disposable gelatin filter (3.0 μm) for sample collection. The air sampler was placed on a tripod at 1.0 m elevation above ground level and was protected by a secured, angled overhead umbrella to prevent direct rain contact. After each air sampling, the gelatin filter was placed in 30 mL of modified Ashdown selective broth and incubated at 37°C, with the broth supernatant plated onto Ashdown agar after 2 and 7 days (*3*). Soil samples were collected at ≈10 cm below the surface. Sterile water (20 mL) was added to 20 g soil and shaken at 220 rpm for 48 h at 37°C. After the soil samples were removed from the shaker and left to stand for 1–2 h, 10 mL of supernatant was placed in 30 mL of modified Ashdown selective broth and incubated at 37°C; the broth supernatant was plated onto Ashdown agar after 2 and 7 days. Suspected *B. pseudomallei* colonies were confirmed by using the *B. pseudomallei*–specific TTS1 real-time PCR, described previously (*5*).

*B. pseudomallei* was cultured from 1 of the 2 air samples and 1 of the 3 soil samples. Multilocus-sequence typing, completed by using standard methods (*6*), confirmed that 2 isolates from each of the positive air and soil samples and the isolate from the patient’s blood culture were all sequence type (ST) 562. To further resolve the relatedness of ST562 isolates, PCR-based, multilocus variable-number tandem-repeat analysis of 4 loci (MLVA-4) was performed as described (*7*) on the 4 environmental isolates, the patient’s blood culture isolate, and isolates from 13 other patients in the Darwin prospective study whose melioidosis was caused by ST562 *B. pseudomallei*. MLVA-4 categorized the 18 ST562 isolates into 3 distinct types: 55 (n = 7), 71 (n = 8), and 168 (n = 3). The isolate from patient 692, all 4 environmental isolates, and isolates from 2 of the 13 other patients were MLVA-4 type 55.

To further define the relatedness of ST562 isolates, whole-genome sequencing was performed on 17 of the 18 ST562 isolates for which MLVA-4 results were available; to reduce duplication and cost, 1 of the 2 *B. pseudomallei* isolates from air samples was not sequenced because it was clonal with the other air sample. Genomic DNA was extracted by using the QIAGEN DNeasy blood and tissue kit (QIAGEN, Chadstone, Victoria, Australia), as described (*7*). Samples were sequenced at Macrogen Inc. (Gasan-dong, Seoul, South Korea) by using HiSeq 2000 (Illumina, San Diego, CA, USA). Genome analysis was performed with SPANDx version 2.3 (*8*) by using the ST562 strain MSHR4388 from the MLST database (http://bpseudomallei.mlst.net/) as the reference genome.

**Figure 2:**
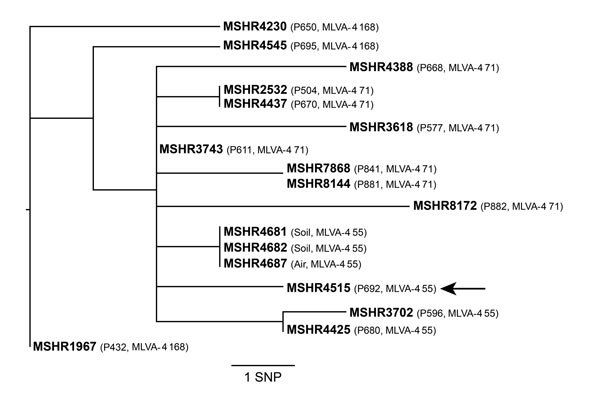
Whole-genome core orthologous single-nucleotide polymorphism (SNP) phylogeny of sequence type 562 *Burkholderia pseudomallei* isolates from a patient with melioidosis and from environmental sampling at the patient’s residence, Darwin, Northern Territory, Australia. MSHR4515 (MLST database identifier, http://bpseudomallei.mlst.net/) was a blood culture isolate from the index patient, identified as patient (P) 692 (P692, arrow). Analysis of isolates from 13 other patients with sequence type 562 are also shown (identifiers begin with P). Comparison of data for NPs and for multilocus variable-number tandem-repeat analysis of 4 loci (MLVA-4) types (shown in parentheses) supports the hypothesis that P692 was infected from environmental *B. pseudomallei* at his residence. Consistency index = 1.

Whole-genome identification of single nucleotide polymorphisms (SNPs), followed by phylogenetic reconstruction by using maximum parsimony in PAUP 4.0b10 (*9*), showed that all ST562 isolates were closely related; only 26 SNPs were observed among all 17 genomes (Figure 2). The air isolate (added to the MLST database as MSHR46817) and the 2 soil isolates (MSHR4681 and MSHR4682) obtained from the environment outside the residence of patient 692 were identical by whole-genome sequencing and differed from the blood culture isolate of patient 692 (MSHR4515) by only 3 SNPs. These genetic similarities support the epidemiologic link between the air and soil *B. pseudomallei* and the patient’s infection.

Epidemiologic data from Australia, Singapore, and Taiwan support the hypothesis that inhalation may replace inoculation as the predominant route of *B. pseudomallei* transmission during severe weather events (e.g., tropical monsoonal storms, cyclones, and typhoons) (*10*–*12*). Similar clinical distinctions between percutaneous and inhalational infections are observed for anthrax, plague, and tularemia (*4*). Animal studies have also shown the potential importance of aerosol inhalation of *B. pseudomallei* with high lethality (*13*,*14*). Inhalational melioidosis is supported by the increasing recognition from computed tomography scanning that enlarged mediastinal lymph nodes are not uncommon in patients with severe melioidosis pneumonia (*4**,**10*). Nevertheless, no direct evidence exists to confirm occurrence of inhalation of *B. pseudomallei* in melioidosis-endemic regions. A recent report from Taiwan documented an air sampling technique that uses a filtration real-time quantitative PCR method to quantify ambient *B. pseudomallei* DNA; high positive rates were found during typhoons (*12*).

## Conclusions

*B. pseudomallei* was recovered from air samples taken outside the residence of a patient with clinical features consistent with inhalational melioidosis. Whole-genome sequencing linked the environmental *B. pseudomallei* to an isolate from the patient’s blood culture. These data provide evidence of aerosolization of *B. pseudomallei* during stormy conditions in an endemic location and strong circumstantial evidence for inhalation of *B. pseudomallei*. The proportion of melioidosis cases resulting from inhalation rather than percutaneous inoculation or ingestion requires further study and is likely to vary substantially by location and season.
